# Long read transcriptome sequencing of a sugarcane hybrid and its progenitors, *Saccharum officinarum* and *S. spontaneum*

**DOI:** 10.3389/fpls.2023.1199748

**Published:** 2023-08-14

**Authors:** Prathima Perumal Thirugnanasambandam, Avinash Singode, Lakshmi Pathy Thalambedu, Selvi Athiappan, Mohanraj Krishnasamy, Sobhakumari Valiya Purakkal, Hemaprabha Govind, Agnelo Furtado, Robert Henry

**Affiliations:** ^1^Crop Improvement Division, ICAR-Sugarcane Breeding Institute, Coimbatore, Tamil Nadu, India; ^2^Indian Council of Agricultural Research (ICAR)-Indian Institute of Millets Research, Hyderabad, Telangana, India; ^3^Queensland Alliance for Agriculture and Food Innovation, The University of Queensland, St. Lucia, Brisbane, QLD, Australia

**Keywords:** *Saccharum officinarum*, Black Cheribon, *Saccharum spontaneum*, Coimbatore accession, Co 11015, progenitors, long read transcriptome, sugar genes

## Abstract

Commercial sugarcane hybrids are derivatives from *Saccharum officinarum* and *Saccharum spontaneum* hybrids containing the full complement of *S. officinarum* and a few *S. spontaneum* chromosomes and recombinants with favorable agronomic characters from both the species. The combination of the two sub-genomes in varying proportions in addition to the recombinants presents a challenge in the study of gene expression and regulation in the hybrid. We now report the transcriptome analysis of the two progenitor species and a modern commercial sugarcane hybrid through long read sequencing technology. Transcripts were profiled in the two progenitor species *S. officinarum* (Black Cheribon), and *S. spontaneum* (Coimbatore accession) and a recent high yielding, high sugar variety Co 11015. The composition and contribution of the progenitors to a hybrid with respect to sugar, biomass, and disease resistance were established. Sugar related transcripts originated from *S. officinarum* while several stress and senescence related transcripts were from *S. spontaneum* in the hybrid. The hybrid had a higher number of transcripts related to sugar transporters, invertases, transcription factors, trehalose, UDP sugars, and cellulose than the two progenitor species. Both *S. officinarum* and the hybrid had an abundance of novel genes like sugar phosphate translocator, while *S. spontaneum* had just one. In general, the hybrid shared a larger number of transcripts with *S. officinarum* than with *S. spontaneum*, reflecting the genomic contribution, while the progenitors shared very few transcripts between them. The common isoforms among the three genotypes and unique isoforms specific to each genotype indicate that there is a high scope for improvement of the modern hybrids by utilizing novel gene isoforms from the progenitor species.

## 1 Introduction

Modern sugarcane hybrids are complex polyploids derived from polyploid progenitor species, *Saccharum officinarum* and *Saccharum spontaneum*. *S. officinarum*, originally found growing in the tropical Papua-New Guinea region, is rich in sugars, due to which it was called noble cane. *S. spontaneum* is a grassy wild species with extensive distribution from Africa to Southeast Asia and the Pacific islands and has a diverse gene pool for adaptability and resistance to biotic and abiotic stresses. *S. officinarum* (2n = 8× = 80, x = 10) has high sugar content of about 18-20 degree Brix and is reported to have been domesticated around 8000 years ago from the wild species *S. robustum* (Pompidor et al., 2021). *S. spontaneum* has various cytotypes, many aneuploid forms (2n = 5× = 40 to 16× = 128; x = 8), and has a sugar content of less than 10 degree Brix (Garsmeur et al., 2018).

The earliest sugarcane breeding and selection program in 1888 in Java, Indonesia, incorporated the disease resistance, hardiness, and tillering capacity of *S. spontaneum* into *S. officinarum* germplasm. The resultant hybrids were repeatedly backcrossed to *S. officinarum* as a recurrent female parent in a process called nobilization (Stevenson, 1965). An important phenomenon called “female restitution” occurs during the crossing with *S. officinarum*, wherein 2n+n and n+n transmission of chromosomes happens in the F1 hybrid and BC1 progeny respectively (Premachandran et al., 2011). The rapid recovery of high sugar commercial types from the interspecific hybridization of *S. officinarum* with *S. spontaneum* is attributed to the transmission of the diploid complement of the *S. officinarum* to the hybrid. The first interspecific hybrid, Co 205, a selection from a cross between *S. officinarum* cultivar Vellai and *S. spontaneum* Coimbatore was developed in India in 1912 while POJ2725 and POJ2878 were developed in Java in 1921(Jackson et al., 2014). These inter-specific hybrids served as the foundation for all the modern hybrids of sugarcane worldwide. Commercial sugarcane hybrids which are derivatives from such hybrids contain the full complement of *S. officinarum* and a few *S. spontaneum* chromosomes imparting the favorable agronomic characters from both the species. Such unequal contribution of each progenitor to the hybrid genome was revealed by genomic *in situ* hybridization (GISH) and fluorescent *in situ* hybridization (FISH) studies, demonstrating that the female parent *S. officinarum* contributed about 80% of the chromosomes to the genome of the hybrids, while the male parent *S. spontaneum* contributed only 10%–20% to the hybrid genome (D’Hont et al., 1996; Piperidis and D’Hont, 2001; Cuadrado et al., 2004; D’Hont, 2005). About 5%–17% of the chromosomes resulted from a recombination of chromosomes from the two parental species. Furthermore, each sugarcane hybrid cross most likely directly reflects the chromosome ratio originally from the two parental species, while phenotypically, the greater the contribution of the wild *S. spontaneum*, the greater the fiber content, hardiness, high tillering and vigor in the hybrid (Matsuoka et al., 2014). The resulting sugarcane hybrid genome is composed of a unique chromosome set (ranging from 100-130), containing up to 12-14 copies of each gene (Piperidis and D’Hont, 2001). The monoploid sugarcane genome is estimated to be 382 Mb in size (Garsmeur et al., 2018) while the polyploid sugarcane nuclear genome is about 10 Gb (D’Hont and Glaszmann, 2001; Hoarau et al., 2001; Le Cunff et al., 2008) The genomes of *S. officinarum* LA Purple and *S. spontaneum* SES208 were explored by earlier studies beginning from 1996 (D’Hont et al., 1996). Recently, genomes of *S. spontaneum*, *S. officinarum* and the hybrid genotype R570 were explored (Zhang et al., 2018; Wang et al., 2022; Zhang et al., 2022). However, the entire polyploid sugarcane genome is not sequenced yet due to the inherent genome complexity resulting from the varied contributions of two to three progenitor genomes (Pompidor et al., 2021), recombination, repetitive content, and alternative splicing (Thirugnanasambandam et al., 2018). The diversity existing in each species of the *Saccharum* complex is so high that sequencing a few genotypes may not truly represent sugarcane. The pan genome concept is very suitable for sugarcane as hybrids show differences in chromosome composition and number, and sequencing just one sugarcane hybrid as a representative might result in missing entire chromosome/chromosomes and their associated genomic information.

For this reason, transcriptomic resources remain a valuable means for unraveling this complex genome. Short read assemblies (Casu et al., 2004; Casu et al., 2007; Figueira et al., 2012; Cardoso-Silva et al., 2014; Park et al., 2015), sugarcane expressed sequence tags (SUCESTs) (Vettore et al., 2001), and *Saccharum officinarum* gene indices (SOGI) (Vettore et al., 2003; Hotta et al., 2010) have formed the basis for initial sugarcane transcriptome studies. However, the short read-based assemblies resulted in chimeric reads and artifacts that do not represent the real transcripts arising from the two different sub-genomes and their recombinant chromosomes. This necessitated the development of sugarcane transcriptome resources based on long read sequencing technology that can capture full length transcripts without the need for assembly. The first reported long read reference transcriptome for sugarcane with 107,598 transcripts was developed from stem, leaf, and root tissues from Australian sugarcane hybrid genotypes (Hoang et al., 2017). The benefits of such long read transcriptomes for sugarcane are enormous. There have been successful experiments in gene editing in sugarcane, leading to modified/altered sugar and biomass compositions (Zale et al., 2016; Kannan et al., 2018; Parajuli et al., 2020; Hussin et al., 2022). These studies were possible as a consequence of the sequencing and identification of the various copies and transcript variants of genes in sugarcane. Here, we show for the first time the sub-genomic origins of transcripts related to the most important traits, sugar and disease resistance, in a modern sugarcane hybrid, in comparison with the founding progenitor species. *S. officinarum* accession Black Cheribon, *S. spontaneum* accession Coimbatore, and the commercial hybrid Co 11015 were chosen for long read transcriptome sequencing using PacBio technology. These progenitors were selected as they occur in the pedigree of the commercial hybrid Co 11015 involving crosses for more than six generations.

## 2 Materials and methods

### 2.1 Plant material, RNA extraction and Iso seq sequencing

**Figure 1:**
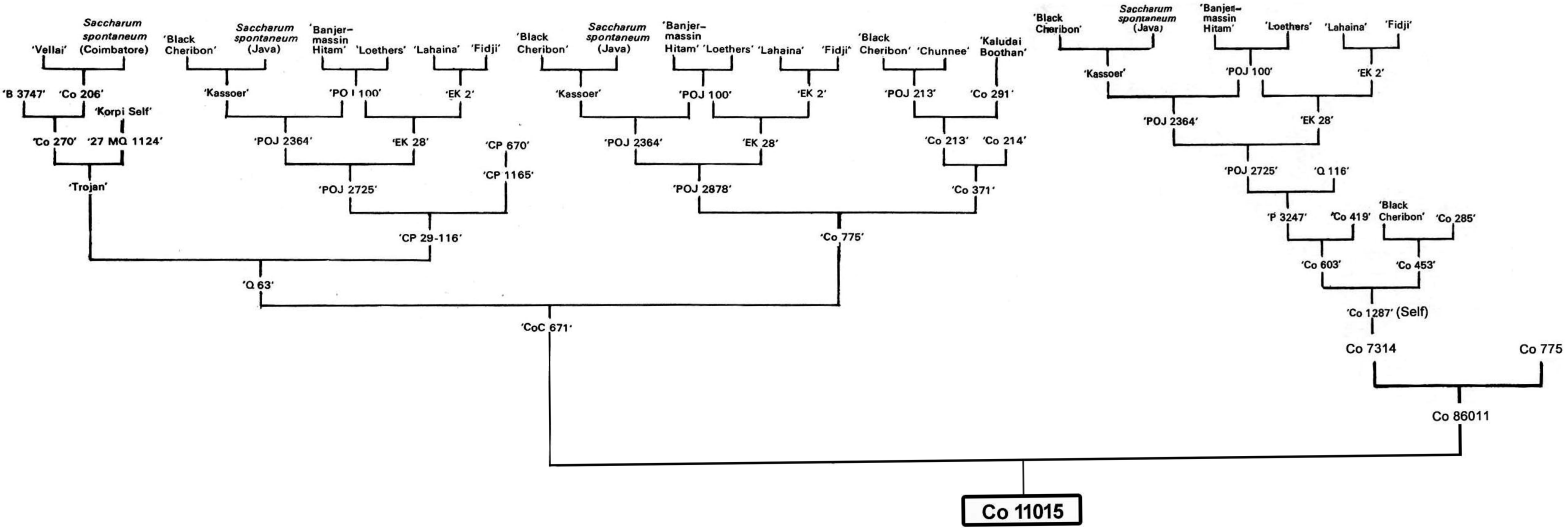
Pedigree map of the sugarcane hybrid cultivar Co 11015.

**Figure 2:**
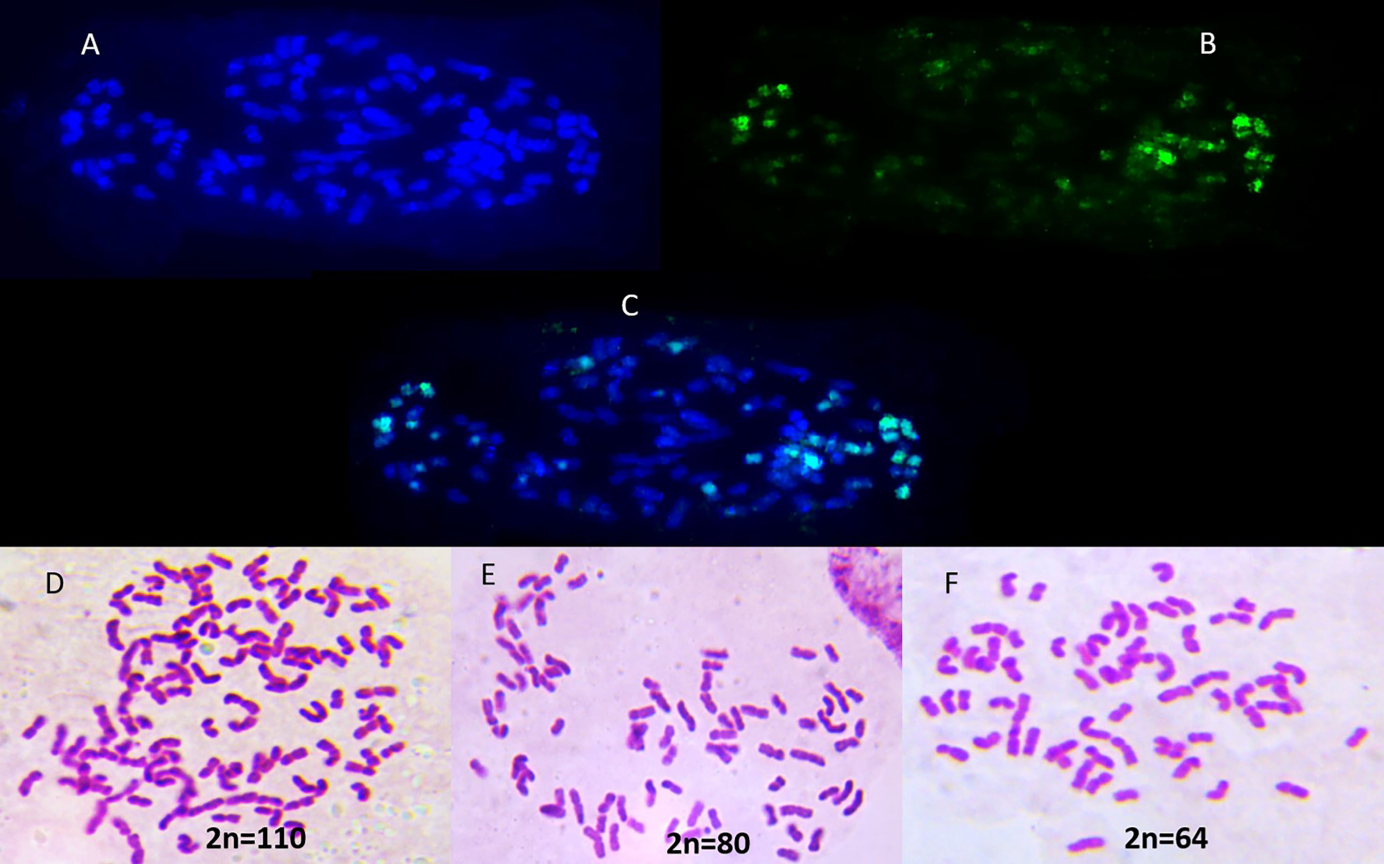
Chromosome composition of **(A)** Sugarcane hybrid Co 11015; **(B)** *S. spontaneum* Coimbatore accession as probe on Co 11015; **(C)** merged Co 11015 and *S. spontaneum* showing fluorescence of *S. spontaneum* (greenish blue); **(D–F)** Chromosome spread of Co 11015; *S. officinarum* Black Cheribon and *S. spontaneum* Coimbatore respectively.

Three sugarcane genotypes, Co 11015 (commercial sugarcane hybrid), Black Cheribon (*S. officinarum*) and *S. spontaneum* (accession Coimbatore), were used in the study. The commercial hybrid Co 11015 was developed at the ICAR-Sugarcane Breeding Institute, Coimbatore (Hemaprabha et al., 2019). The pedigree of Co 11015 is presented in **Figure 1** and the chromosome composition of all the three genotypes is given in the **Figure 2**. Co 11015 is one of the leading cultivars in Southern India and is considered early maturing (can be harvested from the 8^th^ month of planting and has a high sugar content (24 °Brix). Standard crop management practices were followed to raise a healthy crop in the field. The progenitors were selected based on their occurrence in the breeding program of modern commercial hybrids, while Co 11015 was selected on the basis of performance in a field planting of 36 genotypes (data not shown here). Leaf and stem tissues were collected from Co 11015 and *S. spontaneum* planted in the research fields at ICAR-SBI, Coimbatore while *S. officinarum* Black Cheribon leaf and stem tissues were collected from ICAR-SBI Research station, Kannur, Kerala at 12 months after planting. The leaf sample was pooled from three biological replicates while stem samples were collected from top, middle and bottom internodes of three biological replicates and pooled together. The collected samples were immediately frozen in liquid nitrogen and total RNA extraction was performed using the RNeasy Plant Mini Kit (Qiagen, Hilden, Germany) separately for leaf and stem tissues. Total RNA of each sample was estimated by using a Nanodrop 2000 (Thermo Fisher Scientific, Massachusetts, USA) and a Qubit 3.0 fluorometer (Thermo Fisher Scientific, USA) using an RNA HS assay kit (Thermo Fisher #Q32851, Thermo Fisher Scientific, Massachusetts, USA). The integrity of RNA was evaluated on a 1% agarose gel and on an Agilent 2100 Bioanalyzer (Agilent Technologies, California, USA). The RNA was subjected to cDNA synthesis (pooled equimolar from leaf and stem RNA for three genotypes). The amplification of cDNA was done using the NEBNext^®^ Single Cell/Low Input cDNA Synthesis and Amplification Module (New England Biolabs Inc., Massachusetts, USA) in conjunction with an Iso-Seq Express Oligo Kit (Pacific Biosciences, California, USA). Pronex beads (Promega, Wisconsin, USA) were used for the purification of the cDNA before amplification and later for size selection of the amplified product. The library was constructed using the SMRTbell Express template Preparation Kit 2.0 (Pacific Biosciences, California, USA) as per manufacturers’ protocol. The library was purified using Pronex beads (Promega, Wisconsin, USA) and the library size was assessed using a Bioanalyzer (Agilent Technologies, California, USA). About 70 pM of the library was loaded onto one SMRTcell containing 8M ZMW and sequenced in a PacBio Sequel II system in CCS/HiFi mode at the sequencing facility of Nucleome Bioinformatics, Hyderabad, India.

#### 2.1.1 Circular consensus sequence calling and demultiplexing

**Figure 3:**
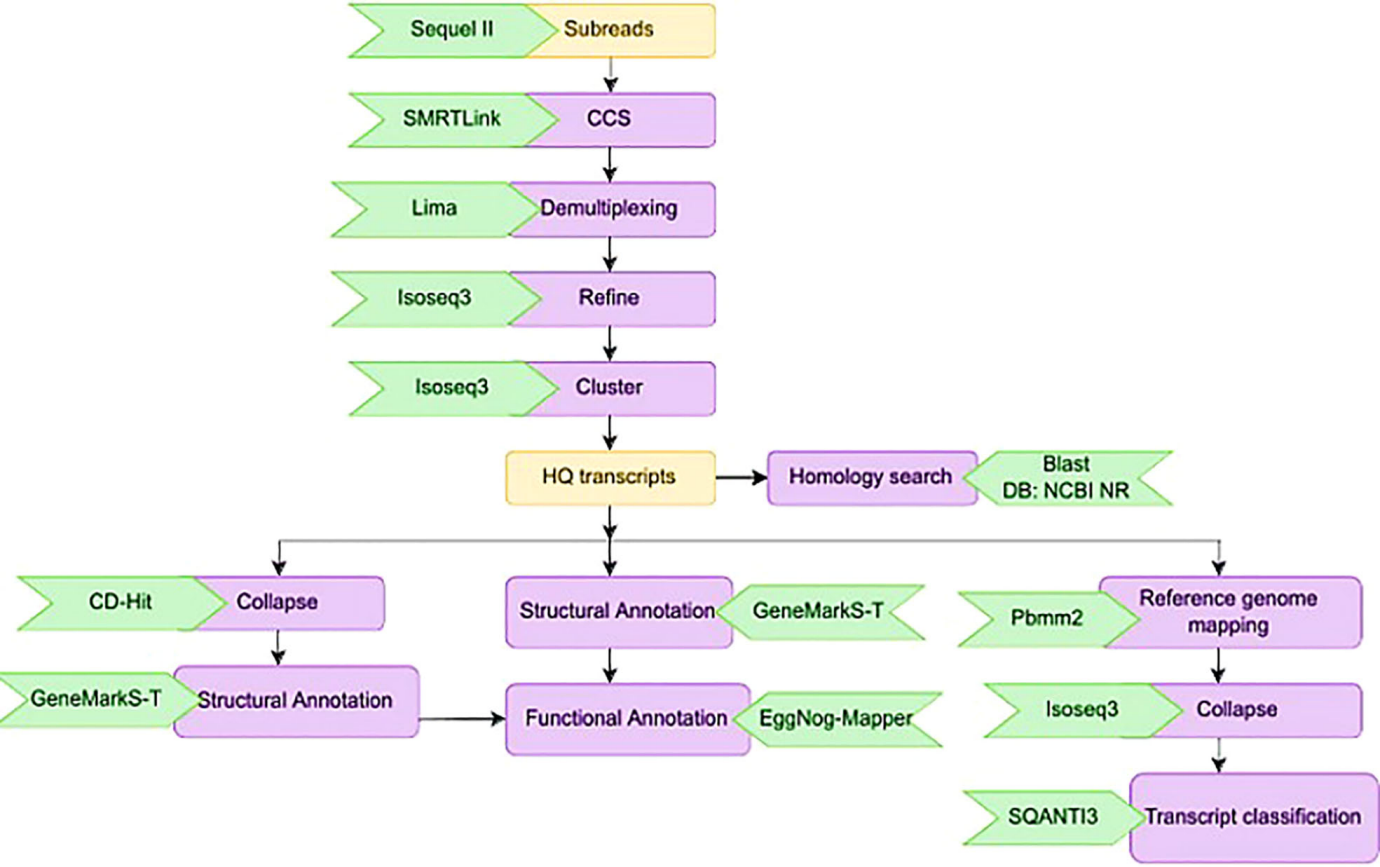
Schematic representation of the bioinformatics work flow for processing HiFi reads from three sugarcane genotypes.

Calling the circular consensus sequence (CCS) is the very first step in processing the Iso-Seq data which was done using the SMRT tool ‘ccs’. This combined multiple sub-reads of the same SMRT bell molecule using a statistical model to produce one highly accurate consensus sequence, also known as a HiFi read. We used Lima, the standard tool to identify barcode and primer sequences in PacBio single molecule sequencing data. The overall workflow for the bioinformatics analysis is given in **Figure 3**. Lima identifies and removes the 5’ and 3’ cDNA barcodes.

##### 2.1.1.1 Refining and clustering

In this step, full-length non-chimeric reads (FLNC) were generated for each sample using the tool ‘isoseq3 refine’, which removes the poly (A) tail and concatemers from the reads. This tool filters for full-length (FL) reads that have a poly (A) tail with at least 20 base pairs and removes the identified tail. The trimmed FL reads are clustered at the isoform level and a consensus is called. Isoseq3 deems two reads to stem from the same transcript if they meet the following criteria: similar transcripts with <100bp 5’overhang, <30bp 3’ overhang, and <10bp gaps. The transcripts with predicted accuracy of ≥0.99 are considered high-quality reads and <0.99 are considered low-quality reads.

##### 2.1.1.2 Reference mapping and collapsing

During library preparation, 5′ RNA degradation products can be formed and are subsequently sequenced. Collapsing is performed to remove the redundant transcript models and especially redundancy caused by reads originated from 5’ degraded RNA. For collapsing the redundant isoforms, clustered high-quality reads were mapped to the reference genome of *Sorghum bicolor* (GCF 000003195.3; https://www.ncbi.nlm.nih.gov/datasets/genome/GCF_000003195.3/) using the pbmm2 tool. These mapped reads were then collapsed using the ‘isoseq3 collapse’ tool.

##### 2.1.1.3 Transcript classification

SQANTI3 (structural and quality annotation of novel transcript isoforms) was used for the classification of the long-read transcriptome. It classifies the transcripts according to their splice junctions and donor and acceptor sites. Transcripts matching a reference transcript at all the splice junctions are labelled as “full splice match” (FSM), and transcripts matching some consecutive, but not at all, are termed “incomplete splice match” (ISM). SQANTI further classifies the novel transcripts of known genes into two categories: “novel in catalogue” (NIC) and “novel not in catalogue” (NNC). Transcripts in novel genes are classified as “intergenic” if lying outside the boundaries of an annotated gene and as “genic intron” if lying entirely within the boundaries of an annotated intron. In addition, the “genic genomic” category encompasses transcripts with partial exon and intron/intergenic overlap in a known gene.

##### 2.1.1.4 *De novo*-based analysis and annotation of high-quality transcripts

To remove the redundancy from the high-quality (HQ) transcripts, we used the cd-hit tool and collapsed the redundant transcripts and obtained the unique transcripts for each sample. The HQ transcripts of each sample were annotated using NCBI Blastn against the NCBI nr database. From the HQ transcripts, protein-coding regions were predicted using the GeneMarkST tool. This uses the heuristic method of initialization of the hidden semi-Markov model and the viterbi algorithm for finding the maximum likelihood parse of the transcript sequence into coding and non-coding regions. Also, it does iterative self-training on sequences. These predicted cds sequences were used for functional annotation with the help of the eggNOG-mapper v2.1 tool.

### 2.2 Comparative genomics

The transcriptomes (HQ transcripts) of *S. spontaneum*, *S. officinarum*, and the sugarcane hybrid Co 11015 were aligned to each other using the alignment and mapping tool in CLC Genomics Workbench v22 (CLC-GWB; Qiagen, Aarhus, Denmark). The percentage of mapping was used to determine the total amount of mapped sequence and the percent identity among the three transcriptomes. The probable sub-genome constitution of the sugarcane hybrid was checked by mapping to both the progenitors. The Co 11015 assembly was compared to the assemblies of *S. officinarum* and *S. spontaneum* by mapping the Co 11015 contigs separately using the two progenitor assemblies as references. The mapping was performed to each reference sequence with a 0.8 length fraction and 0.8 similarity fraction indicating an alignment of two reads with 80% length and similarity coverage. The settings were also varied to 0.9 and 0.9 and 1.0 and 1.0 length and similarity fractions, respectively, to capture the exact transcripts, if any, matching in the reference. The transcriptomes from the three genotypes were aligned to other published genomes of sugarcane and sorghum including *Sorghum* genome v5.1 (https://phytozome-next.jgi.doe.gov/) and sugarcane R570 genome (https://www.ncbi.nlm.nih.gov/genome/10780?genome_assembly_id=386616) with 80% identity and 80% coverage threshold for comparison.

### 2.3 Analyses of sugar and disease resistance genes in the transcriptomes

#### 2.3.1 Sugar genes

The annotated transcriptomes from three genotypes were used for searching for sugar related genes. For this, search terms such as “sugar” and “sucrose” and specific gene lists of the sucrose pathway such as sucrose phosphate synthase (SPS), sucrose phosphate phosphatase, sucrose synthase, and invertases were used for filtering the transcripts.

##### 2.3.1.1 Analysis of sucrose phosphate synthase genes from the three transcriptomes

The SPS genes were filtered from the three transcriptomes and the length distribution of the transcripts was analysed. The transcripts were translated to protein sequences using the Expasy tool (https://web.expasy.org/translate). The protein coding full-length sequences were used for multiple sequence alignment and phylogeny (CLC WB, V22). The tree file was exported in the Newick format and viewed using NCBI Treeviewer. Motif distribution among the SPS transcripts from all three genotypes was found using MEME (https://meme-suite.org/meme/). In addition, sucrose phosphate phosphatase (SPP) and sucrose phosphate translocator (SPT) were also profiled using a similar approach.

##### 2.3.1.2 Disease resistance genes

Similar to the search for sugar genes, for disease resistance, terms such as disease, senescence, -responsive, pathogen, and resistance were used in addition to a list of genes including chitinase, glucanase, and ethylene. The transcripts were filtered accordingly for all three samples, and further analyses were performed.

### 2.4 RNA seq analyses for expression profiling

For expression profiling, RNA Seq reads from sugarcane hybrid genotypes (Mason et al., 2022; Bioproject PRJNA317338) and PRJNA317338 were retrieved from NCBI. The module ‘Expression Analysis using RNA‐seq’ in CLC‐Genome Work Bench (CLC-GWB) version 22 was used. The abundance of each isoform (contributed by SP, BC and Co 11015) was estimated by alignment of the Illumina RNA‐seq data of each sample to the three transcriptomes individually using the RNA‐seq analysis function to have an understanding of the sub-genomic origin of transcripts related to sugar and disease resistance. The reads were aligned to the transcript reads using “one reference sequence per transcript” in the CLC‐GWB’s RNA‐seq package. Normalised expression values were obtained as Reads Per Kilobase of transcript per Million mapped reads (RPKM) and Transcripts Per Million reads (TPM) for further analyses. To identify differentially expressed transcripts, the differential expression (DE) for the RNA‐seq data analysis function in CLC‐GWB was used. The DE analysis in CLC‐GWB uses multi‐factorial statistics based on a negative binomial model (generalised linear model) that considers the various sequencing depths of each sample, facilitating the identification of differentially expressed genes (Mason et al., 2022). A differential gene expression (DGE) table containing the fold changes between samples based on Bonferroni and false discovery rate (FDR) corrected p‐values were used for filtering the expression data.

## 3 Results

### 3.1 Iso seq sequencing of sugarcane progenitors and hybrid

Iso seq sequencing of the samples of *S. spontaneum* (SP), *S. officinarum* (BC), and Co 11015(11) was subjected to initial processing which included generating HiFi reads using the SMRT *ccs* tool. The number of genes and isoforms from each sample and other read statistics are given in the **Table 1**. The majority of the HiFi reads were 2kb to 4kb long. The quality of the 900,000 reads obtained was above Q50 on the Phred scale. The number of HiFi reads after demultiplexing were 679606 in SP, 1076156 in BC and 1268630 in 11. Using ‘Isoseq3 refine’, the high-quality full-length non-chimeric (FLNC) reads generated were found to represent > 95% of total HiFi reads (**Table 2**). These reads were clustered using ‘Isoseq3 cluster’ to create high-quality isoforms with a prediction accuracy of ≥ 0.99. Clusters with ≤ 0.99 prediction accuracy were considered low-quality and were excluded from analyses. Among the three genotypes used in the study, the highest number of clusters was found in BC followed by Co 11015. The high-quality FLNC reads from SP, BC, and 11 were 49908, 119662, and 92500 respectively. The number of splice sites identified in BC transcripts was more than in SP and 11.

**Table 1:** Transcript category statistics based on reference genome.

Category	SP-Leaf-stem		11-Leaf-stem		BC-Leaf-Stem	
FSM	3841		5918		5927	
ISM	2539		3296		11262	
NIC	430		976		1362	
NNC	13268		23589		26692	
Genic Genomic	510		853		517	
Antisense	29		31		58	
Fusion	106		234		307	
Intergenic	95		91		121	
Genic Intron	0		0		1	
Splice Junction Classification						
	SP-Leaf-stem		11-Leaf-stem		BC-Leaf-Stem	
	SJs count	%	SJs count	%	SJs count	%
Known canonical	41960	77.14	49701	72.10	53848	73.74
Known Non-canonical	23	0.04	28	0.04	27	0.04
Novel canonical	1523	2.80	3170	4.60	4494	6.15
Novel Non-canonical	10892	20.02	16030	23.26	14651	20.00

**Table 2:** Details of HiFi reads and clusters obtained for the three genotypes.

Sample	HiFi reads	FLNC reads	% of FLNCs	Clusters of HQ FLNC reads	Unique Transcripts
SP	679,606	663,562	97.6	49,908	40584
11	1,268,630	1,214,852	95.8	92,500	72391
BC	1,076,156	1,069,379	99.4	119,662	99841

### 3.2 Comparative genomics of sugarcane progenitor species and hybrid

**Figure 4:**
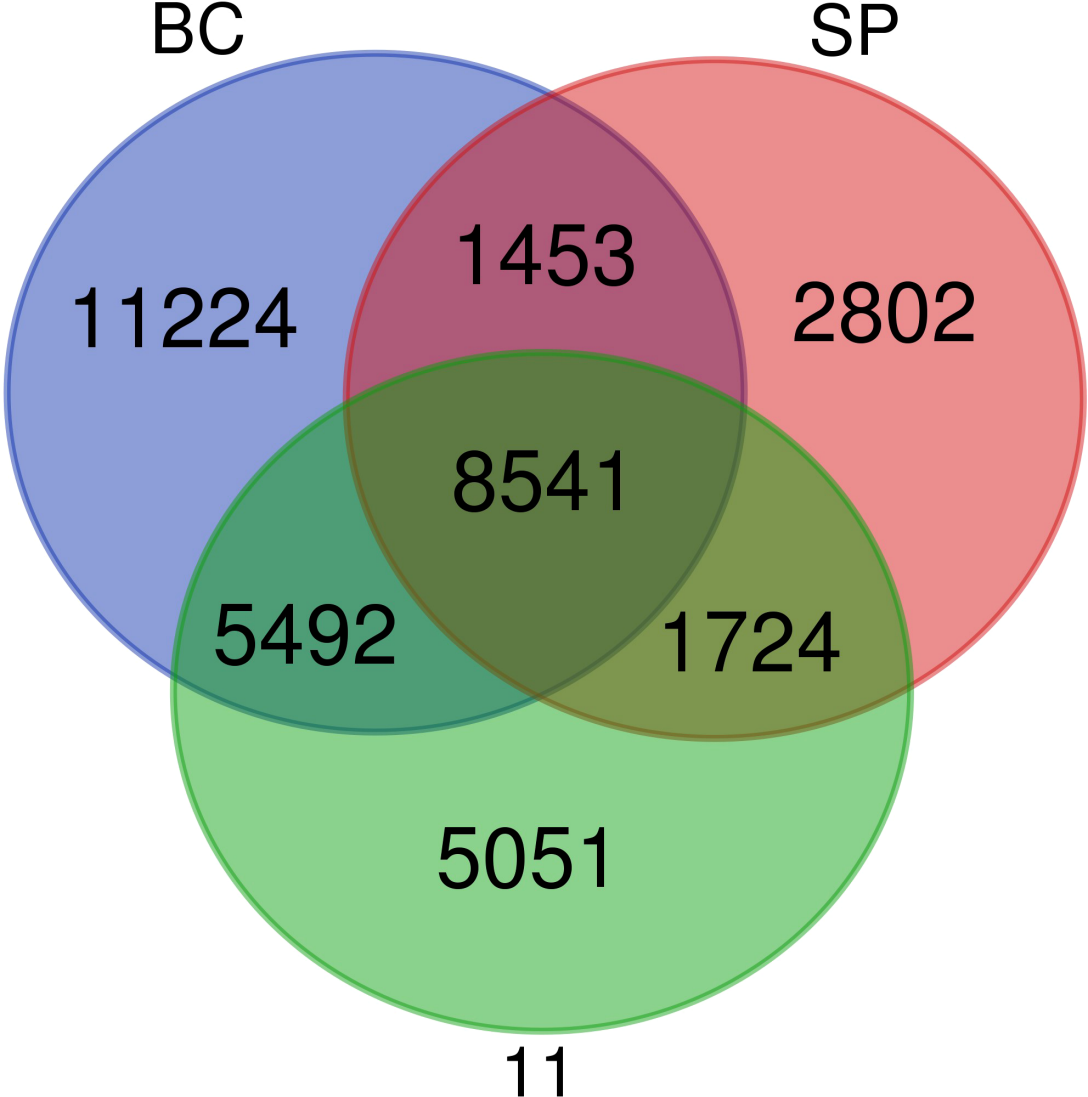
Comparative transcriptome analysis of the sugarcane progenitor species and the hybrid.

The comparative analyses of transcriptomes revealed shared ancestry between *S. spontaneum*, *S. officinarum*, and the sugarcane hybrid Co 11015. The nobilization of sugarcane has harnessed the desirable agronomic and quality traits from both the genomes. Though the sugarcane hybrid Co 11015 and other commercial cultivars are derivatives of BC and SP, significant variation in phenotype and transcript diversity is observed. In our study, the transcriptome of sugarcane hybrid Co 11015 mapped up to 68.7% with *S. spontaneum* and 75% with *S. officinarum*. However, 79% of the Co 11015 transcriptome was mapped on the combined transcriptome of *S. spontaneum* and with *S. officinarum*. A total of 36,287 unique transcripts were found in the combined transcriptome of SP, BC, and 11. The greatest number of transcripts were represented in BC (73.6%) followed by 11 (57.3%) and SP (40.0%). There were 8541 common unique elements in BC, 11, and SP. As expected, the number of common unique elements in SP and 11 were less than the common unique elements found between 11 and BC (**Figure 4**). Comparative transcriptome analysis between the three genotypes and the reference genome of *Saccharum* hybrid cvr SP80-3280 showed a similarity of 36.4%, 40.4%, and 34% with 11, BC, and SP, respectively. Mapping with the reference *S. spontaneum* genome assembly (accession number GCA_022457205.1_ASM2245720v1) was 39.8% for 11, 40.1% for BC, and 43.4% for SP. When *Saccharum* the hybrid R570 assembly MTP (accession number GCA_900465005.1) was used for mapping, a mapping percentage of 30.8% for 11, 31.5% for BC, and 29.5% for SP was recorded. With the *S*. *officinarum* LA Purple reference genome (GCA_020631735.1_ ASM2063173v1), 51.9% of BC, 47.2% of 11, and 29.0% of SP mapped. The results of reference genomes and their mapping percentage with SP, BC, and 11 are given in the **Table 3**.

**Table 3:** Mapping results of SP, BC and 11 with different reference genomes.

S.No	Organism	Reference	Percentage mapping(%)		
			BC	SP	11
1.	*Sorghum bicolor* (cvr: BTx623)	GCA_000003195.3	26.32	28.96	26.61
2.	*S. spontaneum* (Isolate Np-X)	GCA_022457205.1	40.10	43.39	39.81
3.	*S*. *spontaneum* (AP85-441)	GCA_003544955.1	40.64	44.01	40.63
4.	*S*. *officinarum* (LA purple)	GCA_020631735.1	51.90	28.96	47.22
5.	Saccharum hybrid (cvr R570)	GCA_900465005.1	31.48	29.35	30.82
6.	Saccharum hybrid (cvr SP80-3280)	GCA_008692665.1	47.17	41.59	43.81
7.	Saccharum hybrid (cvr SP80-3280)	GCA_002018215.1	40.42	33.99	36.41
8.	Saccharum hybrid (cvr SP80-3280)	GCA_009173535.1	2.26	2.03	2.25
9.	Saccharum hybrid (cvr CC_01_1940)	GCA_020102875.1	43.71	40.62	42.21
10.	Sugarcane SUGIT transcriptome	GFH_J01000000	62.11	62.25	57.21

### 3.3 Transcript diversity in the hybrid and the progenitor genomes

**Figure 5:**
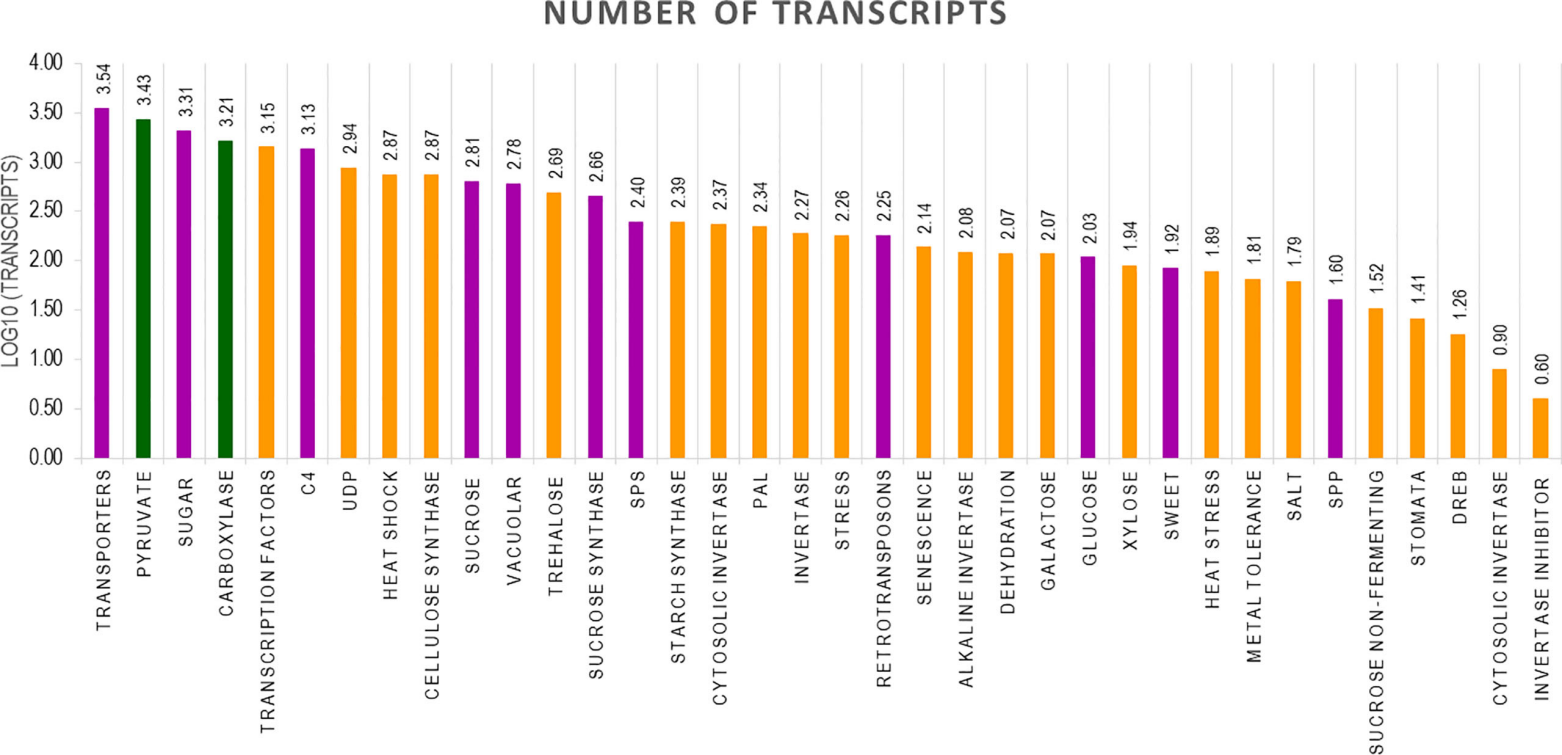
Number of transcripts in 37 groups. The highest number of transcripts in three genotypes are depicted in different colours. Green bar indicates SP, Purple bar indicates BC and Yellow bar 11.

The final Iso seq transcripts were annotated to assign gene function. The total number of transcripts in BC was more (119,662) than in 11 (92,500) and the least was in SP (49,908). The sequences were blasted in the NCBI database. Most of the transcripts matched *Miscanthus* spp. which is a genus related to *Saccharum*. The other transcripts found hits in 15 other genera, among them the most frequent hits were to *Sorghum* sequences in the database. Most of the hits came from C4 members of the Panicoideae sub-family (**Supplementary Figure 1A–C**). The annotated transcripts in SP, BC, and 11 were filtered using functional keywords such as “sucrose”, “sugar”, and “transporters”. Likewise, 37 groups were identified using the filtering keywords that were related to important functions in the plant system. Variation in the number of transcripts related to a function were filtered and counted in the three genotypes. Overall, the most abundant transcripts were related to sugar, transporters, and pyruvate (**Figure 5**). Transcripts related to pyruvate and carboxylases were greater in SP than in BC and 11. In BC, the transcripts related to sucrose pathways in general, i.e., SPP, sucrose synthase, sugar SWEETs, and retrotransposons, were high. Co 11015 showed a higher number of transcripts related to uridine diphosphate (UDP); transcription factors; invertases; other sugars such as xylose, trehalose, and galactose; and stress responsive genes related to DREB, heat, senescence, and dehydration. Lignin related transcripts were absent in BC while “mannose” related transcripts were more abundant in BC. Among the transcripts related to photosynthesis, 11 showed more abundance than SP and BC. Another interesting observation was that 11 had the greatest number of invertases along with the concomitant expression of invertase inhibitors transcripts whereas transcripts for invertase inhibitors were not found in BC or SP.

### 3.4 Analyses of sugar and disease resistance genes

**Figure 6:**
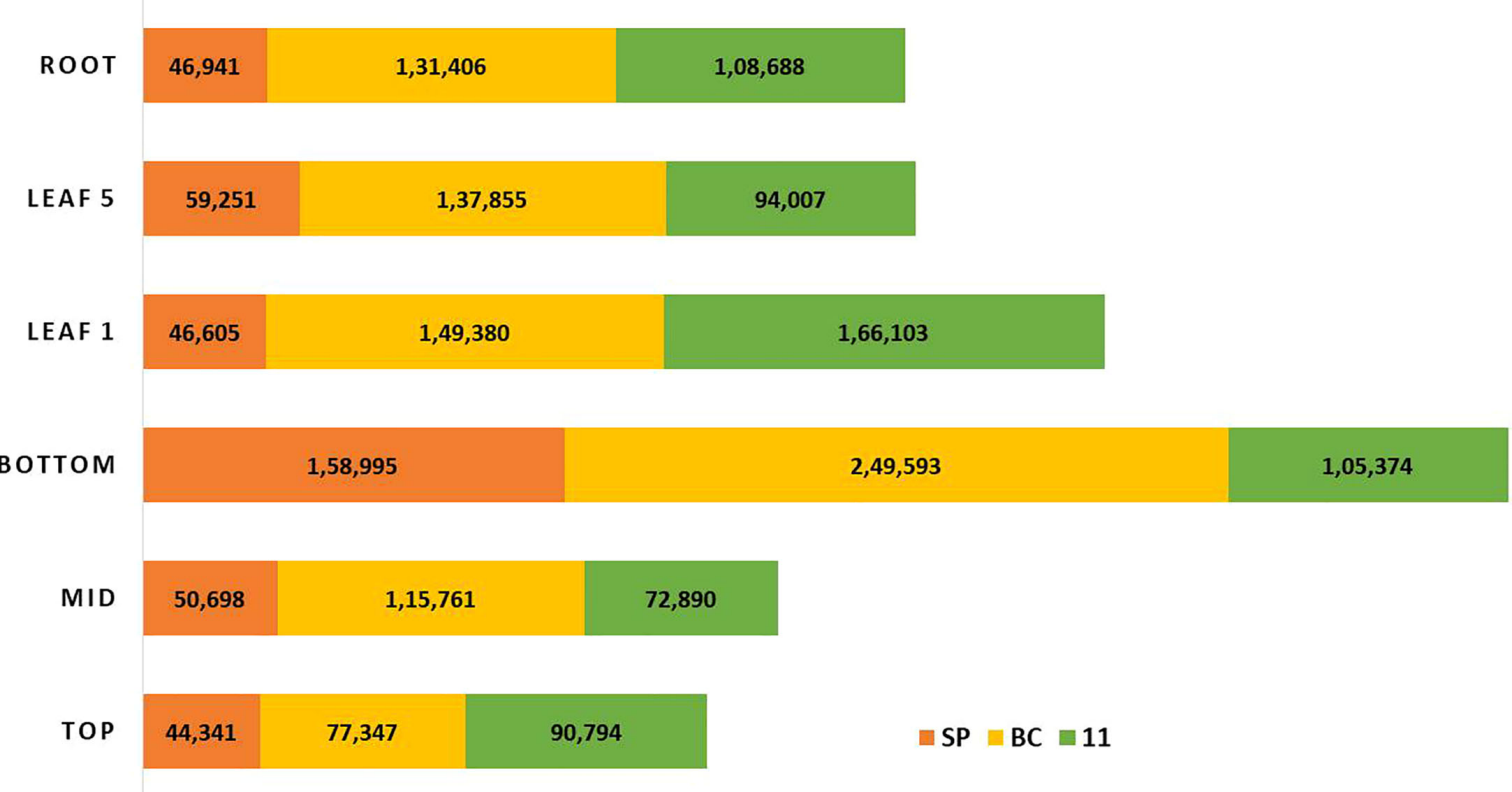
The overall expression pattern of transcripts originating from the three genotypes; red-*S. spontaneum*; yellow-*S. officinarum*; green-*Saccharum* hybrid in the sugarcane tissues, leaf 1 and 5, and the top, middle and bottom tissues of the culm and root.

There were 231, 1792, and 482 genes related to sugar and sucrose in SP, BC, and 11 respectively. Transcripts for other sugars such as trehalose, mannose, and xylose were also checked (**Figure 6**). The number of transcripts related to each component in the three transcriptomes is presented in **Supplementary Tables 1A–C**. The sugar genes were found to be higher in BC than in 11 and SP. Most of the sugar genes were related to transporters. The transcripts related to each of the genes/enzymes of the pathway are presented in **Supplementary Table 1C**.

#### 3.4.1 Analysis of sucrose phosphate synthase genes from the three transcriptomes

**Figure 7:**
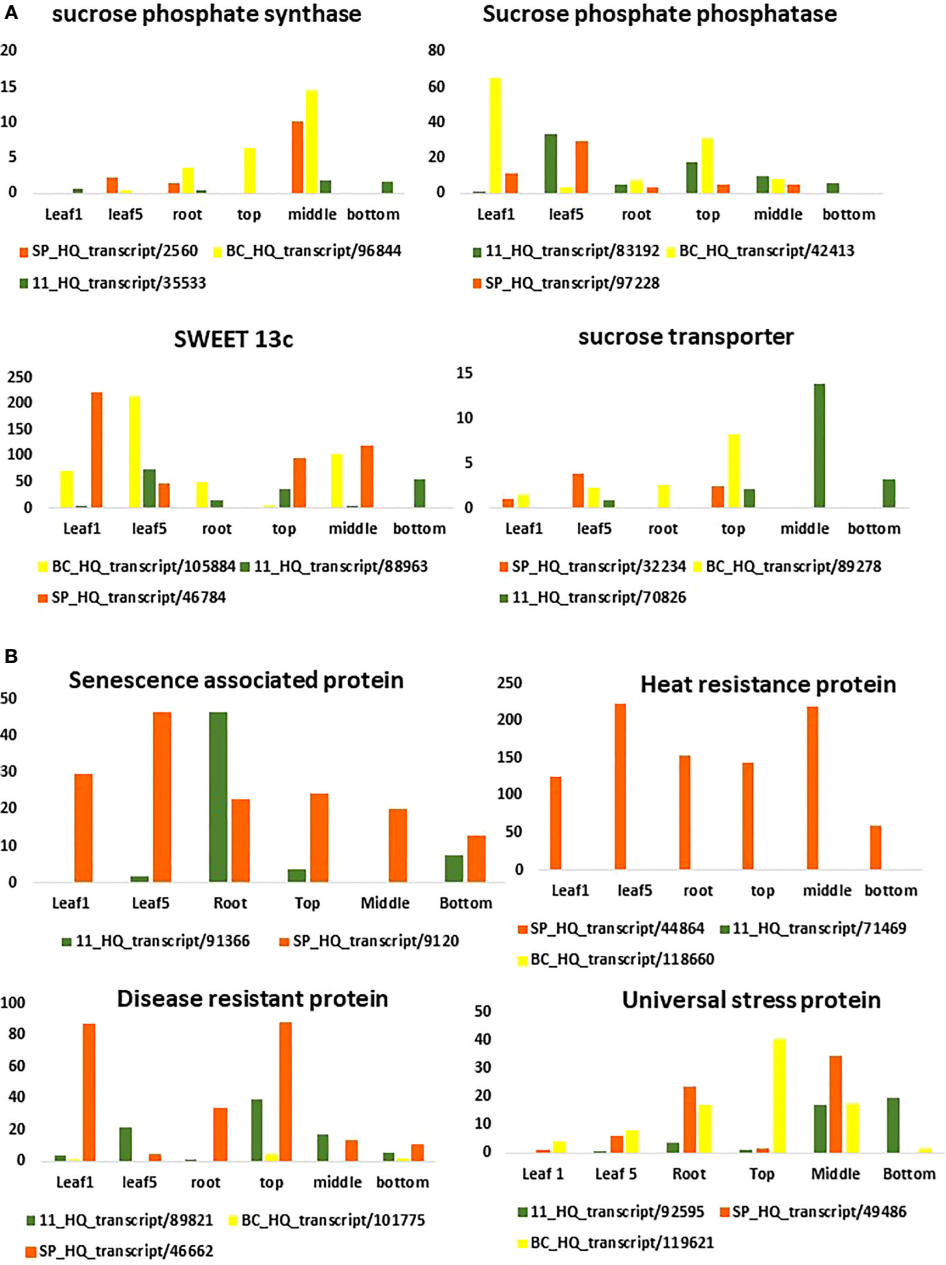
Expression pattern of sugar **(A)** and disease related genes **(B)** in different tissues using the three transcriptomes as references for RNA seq analysis.

**Figure 8:**
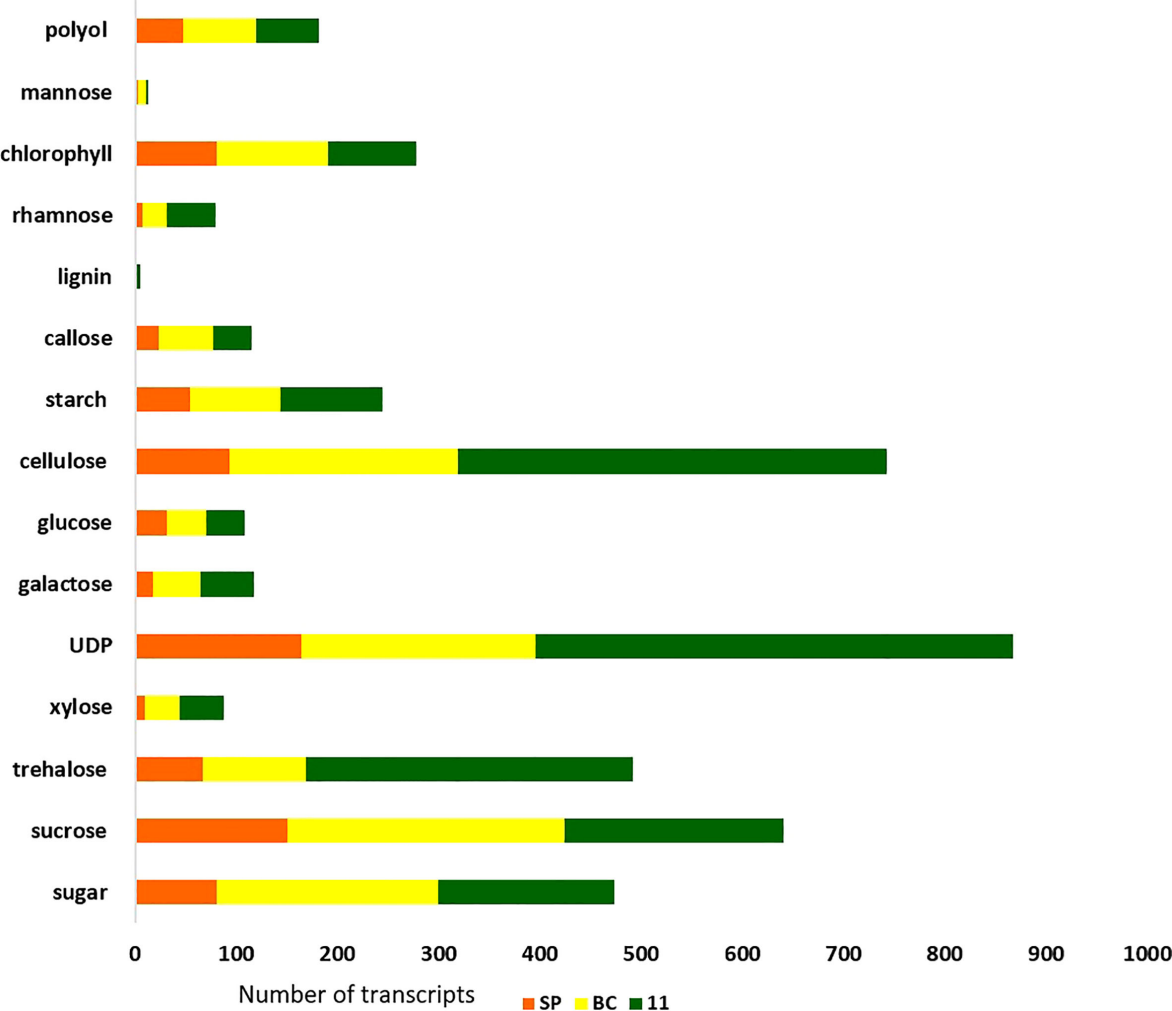
Sugar and other metabolite related transcript expression in the three genotypes: 11, SP, and BC.

**Figure 9:**
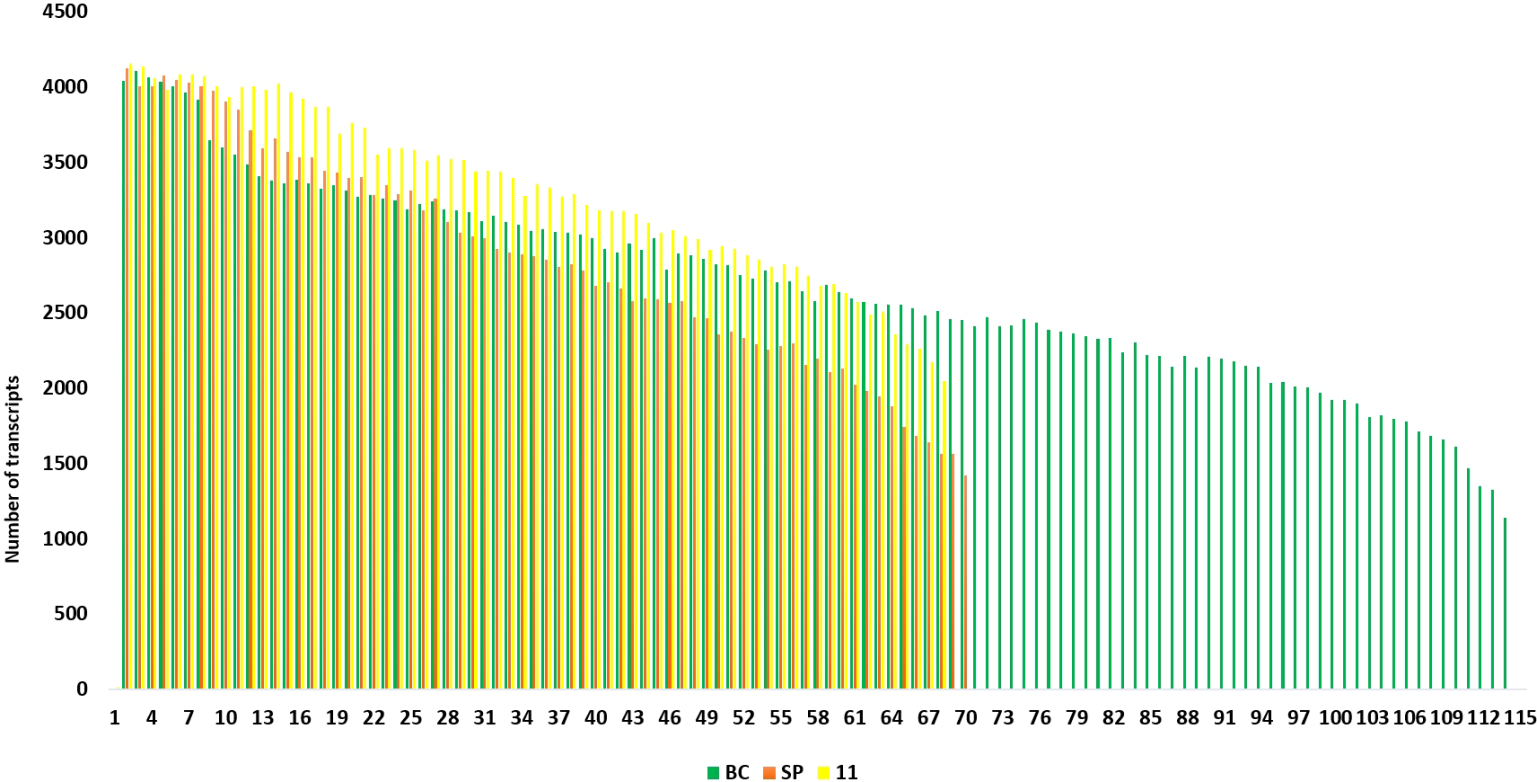
Transcript length distribution for sucrose phosphate synthase genes in *S. spontaneum* (red); *Saccharum* hybrid Co 11015, and *S. officinarum* (green).

The SPS genes were filtered from the three transcriptomes and the length distribution of the transcripts was visualized (**Figure 7**). The translated full length protein sequences were used for multiple sequence alignment, phylogeny, and motif distribution analysis (**Figure 8**; **Supplementary Figures 2A–F**). In total, there were 67, 113, and 69 SPS transcripts from 11, BC, and SP respectively. The total transcripts were further classified into four categories: A, B, II, and SPS. The composition of SPS transcripts from each genotype is shown in **Figure 9**. SP had a higher number of SPSB, whereas 11 had a higher number of SPSA while BC had all four categories almost equal in proportion. In the RNA seq expression analysis with SP and BC, SPSA was expressed in the middle tissues of the culm while with 11, there was no expression of SPSA transcripts. SPSB was expressed at high levels in the leaf tissues of all three transcriptomes (**Supplementary Figures 3A–D**). The transcript details for SPT and their expression profiling are shown in **Supplementary Figure 3E**.

### 3.5 RNA seq analysis using the hybrid and progenitor transcriptomes

**Figure 10:**
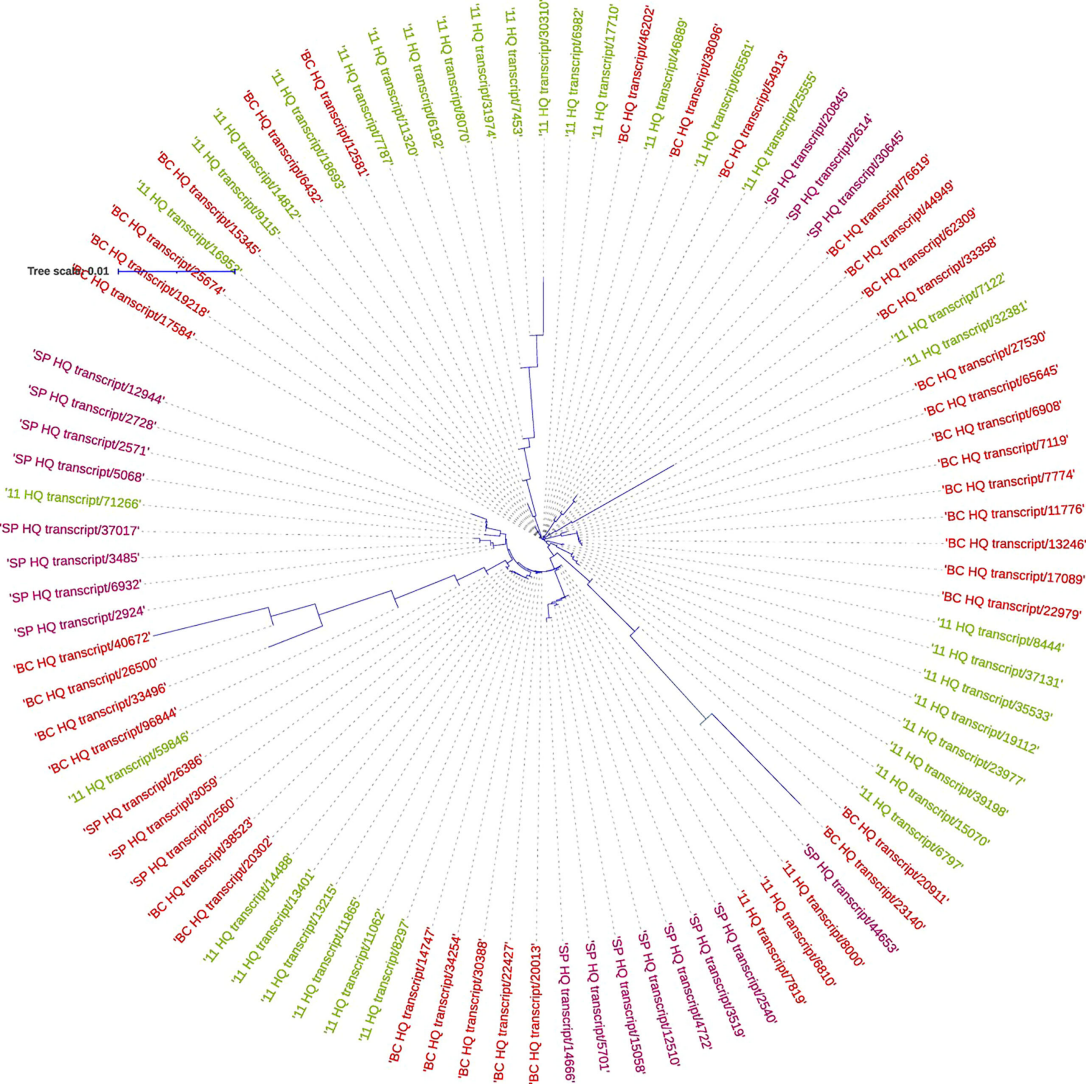
Phylogenetic analysis of sucrose phosphate synthase genes in *S. spontaneum* SP (purple), *Saccharum* hybrid Co 11015 (green), and *S. officinarum* BC (red).

**Figure 11:**
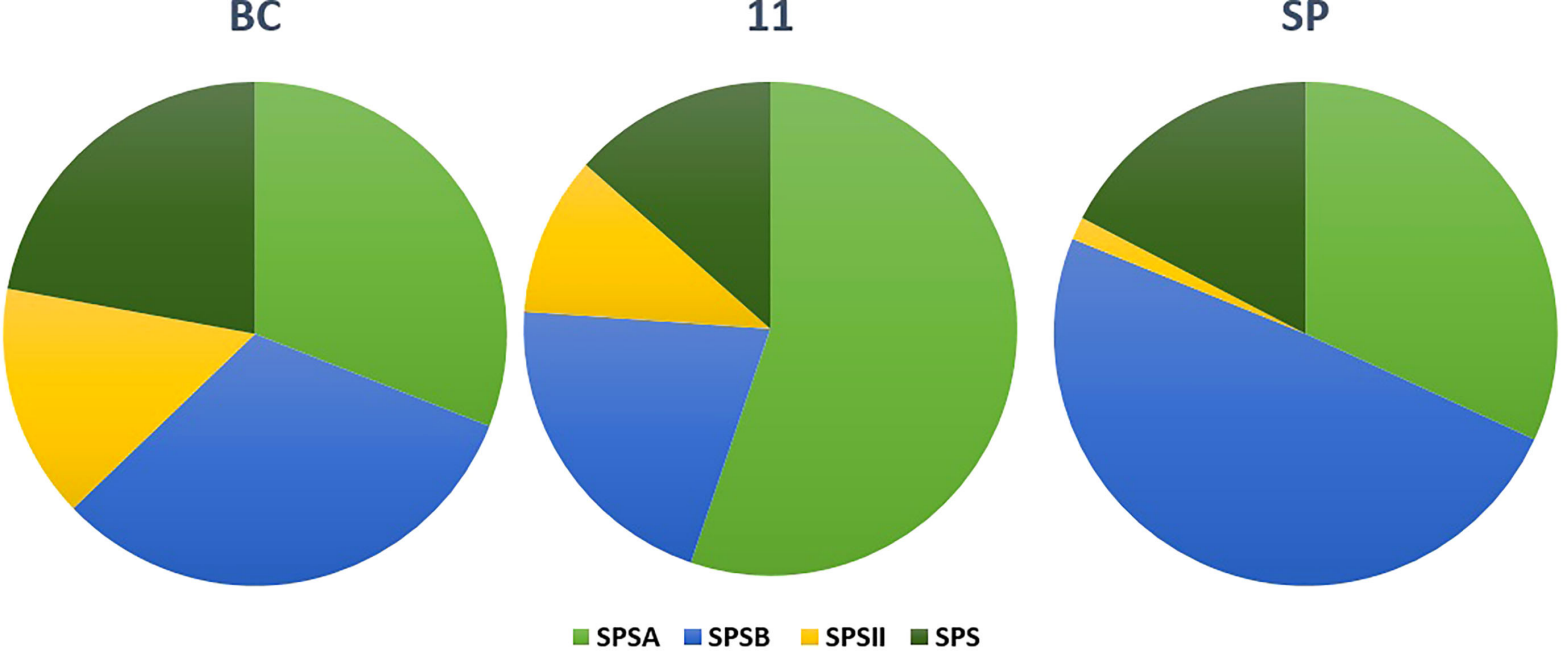
Composition of sucrose phosphate synthase (SPS) transcripts in the three genotypes: SP, BC, and 11.

RNA Seq reads derived from leaf 1, leaf 5, root, and culm samples from the top, middle, and bottom of sugarcane hybrid genotypes were used for profiling spatial expression bias from sub-genomes and the hybrid. The proportion of each tissue-expressed transcripts originating from the three transcriptomes and the results are presented in **Figures 10** and **11**. Similarly, RNA seq reads from stressed and control samples for water stress revealed that the higher expressions of stress related transcripts in the stressed samples were from SP and 11 compared to BC (**Supplementary Figures 4A–E**).

## 4 Discussion

Modern sugarcane hybrids are complex polyploids derived from inter-specific hybridization involving two progenitor species, *S. officinarum* and *S. spontaneum*. Sugar content and disease resistance were the characteristic traits for which the modern hybrids were selected over generations of breeding programs. Knowledge of the share of the progenitors in manifesting higher genetic gains in terms of these two traits would help in widening the gene pool further in developing future-ready cultivars. The long read transcriptomes of a modern hybrid, Co 11015, and its progenitors, *S. officinarum* Black Cheribon and *S. spontaneum* Coimbatore, were developed and dissected for sugar and disease resistance. *S. officinarum* Black Cheribon served as a common parent in almost all the sugarcane breeding programs and would probably be the most common ancestor for all the sugarcane cultivars being grown around the world. The proportional genome content of *S. officinarum* in the progenies seems to determine the sucrose synthesis and accumulation potential of the genotype. *S. spontaneum* clones are wild weedy plants, which are mainly non-cane forming types, and are therefore not used as immediate parents for commercial breeding purposes. Co 11015 is an early maturing variety developed at ICAR-Sugarcane Breeding Institute, Coimbatore. This genotype was a selection from the cross between high sucrose clones, CoC 671 and Co 86011, for which POJ 2725 is a common parent (**Figure 1**).

Iso seq sequencing of *S. spontaneum* (SP), *S. officinarum* (BC), and Co 11015 (11) resulted in 679606, 1076156, and 1268630 HiFi reads in SP, BC, and 11 respectively, mostly in the range of 2kb to 4kb in length. The FLNC reads represented more than 95% of the HiFi reads out of which only the high-quality isoforms were used for further analyses. There were 49908, 92500, and 119662 clustered high-quality (HQ) reads in SP, 11, and BC, respectively. Among the three genotypes used in the study, the highest number of clusters as well as splice junctions were found in BC, followed by 11, suggesting that BC has a more complex genome than the hybrid. The comparative analyses of transcriptomes revealed that sugarcane hybrid Co 11015 mapped up to 68.7% with *S. spontaneum* and 75% with *S. officinarum.* When the three transcriptomes were combined, 36,287 transcripts were found to be unique. BC had a major share (73.6%) of the unique transcripts followed by 11 (57.3%) and SP (40%), further suggesting its complexity. The unique transcripts in BC might have undergone the process of unconscious negative selection for those genes during the course of selection of the intermediate parents that ultimately gave rise to Co 11015. This might be one of the reasons for the low mapping percentages of the progenitor transcripts with the hybrid. The unique transcripts in the hybrid might have partly originated from the other *S. officinarum* and *S. spontaneum* genotypes which were part of its ontogeny such as Loethers, Banjermassin Hitham, chunnee, and *S. spontaneum* Java. Comparative transcriptome analysis of the three transcriptomes with published reference genomes indicated a shared common ancestry with cultivars such as R570 and SP80-3280.

The hybrid’s and the progenitor’s transcriptomes were analyzed for overall gene expression in general and genes for the important traits sugar content and disease resistance, in particular. The transporters category was the largest in all the three genotypes, with BC having higher number of transcripts compared to 11 and SP. Second was the transcription factor (TF) category with 11 having the highest number of transcripts than BC and SP. Transcripts for trehalose, UDP, phenyl ammonia lysase, cellulose, heat, stress, senescence, starch, pyruvate, metal and salt tolerance, drought, invertases, and invertase inhibitor were higher in 11. In fact, the transcripts for trehalose are higher than the transcripts for sucrose in 11 (**Supplementary Tables 1A–C**). The large number of UDP and photosynthesis related transcripts in 11 suggests the availability of substrate for many cellular processes translates into higher sugar and biomass in the hybrids. Another interesting observation was that the retrotransposons were lower in 11 than BC, suggesting that the hybrid is less complex than the parental genome of BC which is also corroborated by the lower number of splice junctions and total number of unique transcripts observed in 11 compared to BC (**Table 2**). SP has a very low number of retrotransposons compared to BC and 11.

For studying the gene expression pattern for sucrose, sucrose phosphate synthase (SPS) was selected for a detailed analysis. It is a key regulatory enzyme involved in sucrose biosynthesis. This enzyme catalyzes the transfer of a hexosyl group from UDP glucose to D-fructose 6-phosphate to form UDP and D-sucrose-6-phosphate. SPS is critical in the accumulation of sucrose because the reaction is irreversible. Sucrose synthase, on the other hand, involves a reversible reaction that allows sucrose to engage in a variety of metabolic activities, including tissue formation, material storage, and plant cell metabolism (Huber, 1983). SPS genes were categorized into three distinct families (A, B, and C) with different evolutionary histories in dicots (A family) and monocots (B family) (Langenkamper et al., 2002). Expression studies on *S. officinarum* and *S. spontaneum* have shown that *S. officinarum* had a higher expression of SPS A and SPS B than *S. spontaneum* (Ma et al., 2020). We observed a large number of SPS B in SP and SPS A in the sugarcane hybrid while BC had all the categories in equal proportions. The physiological relevance of these categories is not yet known and needs further study. However, it can be observed from the tissue specific expression study that SPS B is expressed in leaf tissues while SPS A shows higher expression levels in the culm tissues (**Supplementary Figures 3A, B**), suggesting SPS A could have an important role in stem sugar accumulation. In the case of disease resistance, the expression profiling of dehydrins, heat shock proteins, abscisic stress-ripening, aquaporin, senescence associated protein, etc. clearly show a higher contribution from SP than BC and 11 of hybrid genotypes under stress (**Supplementary Figures 4A–E**). A similar trend was observed in the transcriptomes with a higher number of transcripts in the stress, heat, and senescence categories in SP and 11 than BC. It must be noted that although the total number of transcripts in SP was only 49908 compared to 119662 (BC) and 92500(11), it showed an equal or higher number of transcripts for stress, senescence, heat, etc. as that of 11 and BC. (**Supplementary Table 1A**).

An interesting aspect of the inter-specific hybrids explored by previous studies is that *S. spontaneum* is a potential source of genes for sugar content. Studies on sugar composition from the *S. spontaneum* genotypes in the world collection in Miami, Florida, revealed positive alleles for sugar content (Tai and Miller, 2001). *S. spontaneum*-specific polymorphic markers for sugar content were identified and used for tagging positive *S. spontaneum* alleles for introgression into commercial sugarcane genotypes (da Silva et al., 2007). Although the sucrose accumulating potential of *S. spontaneum* accessions can hardly be estimated based on their performance *per se*, progeny performance can be taken as an indirect measure of the breeding value of the parent. This principle formed the basis for developing linkage maps and identifying the genomic regions governing sucrose content in *S. spontaneum* (Ming et al., 2001; Aitken et al., 2005; da Silva and Bressiani, 2005). However, these studies were limited by the number of molecular markers and the genotypes that were used in the experiments. In this study, SP was found to have a large number of transcripts for pyruvate carboxylase, sucrose transporters, trehalose phosphate phosphatase, acid invertase, and sucrose non-fermenting kinases, and, for some genes, there was similarity in the number of transcripts with 11 (**Supplementary Figures 5A, B**). *S. spontaneum* could also be speculated to be a source of sugar genes due to the presence of SPS B and a large number of transcripts related to pyruvate and trehalose, but this needs further study.

The transcriptomes from the progenitors described above and from the hybrid very clearly indicate the potential of such resources in understanding the gene regulation for important traits at the molecular level. It also suggests that the hybrid transcriptome of 11 has evolved its own genetic makeup apart from the mixture of genomes from the progenitors (**Figure 8**). From the expression profiling experiments, it is clearly evident that the genes for vigor and adaptation to various biotic and abiotic stresses in the hybrid might have been contributed by *S. spontaneum* while the genes for sugar and transporters were from *S. officinarum*. Studies on each and every gene set would be exhaustive, highly informational, and would provide us clues of the inheritance pattern of traits from the crosses. As observed from the transcripts, BC probably has a much more complicated genome structure than the hybrids, however, the hybrid seems to have a higher degree of sophistication in terms of transcription factors, biomass related genes, invertases, and the transcripts related to several sugars other than sucrose.

Unlike several other polyploid crops such as Brassica or wheat, sugarcane hybrids did not involve diploid progenitors. Diploid progenitors are often used as model systems for studying the gene expression bias/dominance and genomic changes in the formation of hybrids resulting from polyploidization. Extensive alterations occur during the merger of diverged polyploid genomes at each level of crossing, resulting in the formation of novel transcriptome networks. The extent of homolog expression bias changes over generations, from the initial sub-genome merger through to the incorporation of new genomes until the hybrid is selected. Novel transcripts, gene networks, and regulatory elements can emerge in the hybrids, however, the progenitors, which themselves are polyploids, may still hold important genes and perform similarly or equivalently to hybrids. There are no studies on the expression level dominance of sub-genomes in sugarcane hybrids due to limitations in the currently available genomics tools. However, to a certain extent, the direction of expression level dominance could be observed from the transcriptome of sugarcane hybrid Co 11015 in comparison with the transcriptomes of the progenitors.

## 5 Conclusion

The gene pool of sugarcane hybrids needs to be widened with more input from the valuable germplasm available from around the world to meet the fresh demands of agriculture today. We have developed transcriptome resources from progenitor species and a hybrid for a comparative study to look deeper into the parental materials for new perspectives in terms of their contribution to the hybrid and to improve the traits of interest in a more precise manner. The long reads offer a great advantage compared to short reads, particularly, as there is no assembly involved. This facilitates the identification of the exact isoform of a gene that may help in modifying a trait, for example, to increase the sugar content, and, in the future, the exact time of its expression. Further studies on more genotypes and their sequence information will provide a comprehensive understanding of the sugarcane genome complexity and gene regulation.

## Data availability statement

The datasets presented in this study can be found in online repositories. The names of the repository/repositories and accession number(s) can be found below: https://www.ncbi.nlm.nih.gov/ and https://doi.org/10.6084/m9.figshare.21974702. NCBI Sequence BioProject ID PRJNA479814, study Accession Number SRP152893 and PRJNA317338, study accession number SRP075950.

## Ethics statement

Sugarcane commercial genotypes and germplasm collection were collected from the field planting at ICAR-Sugarcane Breeding Institute, Coimbatore, Tamil Nadu, India. No ethics approval was required for the conduct of experiments in this study.

## Author contributions

PT conceived and designed the experiments. PT, MK, and LT collected the samples. PT, AS, SA, and SP conducted analyses. PT prepared the first draft. PT, RH, AF, AS, and HG critically revised the manuscript. All authors contributed to the article and approved the submitted version.
